# Protective Effect of Fat Extract on UVB-Induced Photoaging *In Vitro* and *In Vivo*

**DOI:** 10.1155/2019/6146942

**Published:** 2019-08-18

**Authors:** Mingwu Deng, Yuda Xu, Ziyou Yu, Xiangsheng Wang, Yizuo Cai, Hongjie Zheng, Wei Li, Wenjie Zhang

**Affiliations:** Department of Plastic and Reconstructive Surgery, Shanghai 9th People's Hospital, Shanghai Jiao Tong University School of Medicine, Shanghai Key Laboratory of Tissue Engineering, National Tissue Engineering Center of China, Shanghai, China

## Abstract

**Background:**

Nanofat can protect against ultraviolet B- (UVB-) induced damage in nude mice. Fat extract (FE) is a cell-free fraction isolated from nanofat that is enriched with a variety of growth factors.

**Objective:**

To determine whether FE can protect against UVB-induced photoaging in cultured dermal fibroblasts and in nude mice.

**Method:**

For the *in vitro* study, human dermal skin fibroblasts were pretreated with FE 24 h prior to UVB irradiation. Generation of reactive oxygen species (ROS) was analyzed immediately following irradiation, while cell cycle analysis was performed 24 h after UVB irradiation. Senescence-associated *β*-galactosidase (SA-*β*-gal) expression, cell proliferation, and expression of glutathione peroxidase 1 (GPX-1), catalase, superoxide dismutase-1 (SOD-1), SOD-2, and collagen type 1 (COL-1) were investigated 72 h after UVB irradiation. For the *in vivo* study, the dorsal skin of nude mice was irradiated with UVB and mice were then treated with FE for 8 weeks. The thickness of the dermis, capillary density, and apoptotic cells in skin tissue sections were investigated after treatment. The expression of GPX-1, catalase, SOD-2, SOD-1, and COL-1 in the tissue was also measured.

**Result:**

FE significantly increased cell proliferation and protected cells against UVB-induced cell death and cell cycle arrest. FE reduced ROS and the number of aged cells induced by UVB irradiation. FE promoted the expression of COL-1 and GPX-1 in cultured dermal fibroblasts. FE treatment of UVB-irradiated skin increased dermal thickness and capillary density, decreased the number of apoptotic cells, and promoted the expression of COL-1 and GPX-1.

**Conclusion:**

FE protects human dermal fibroblasts and the skin of nude mice from UVB-induced photoaging through its antioxidant, antiapoptotic, and proangiogenic activities.

## 1. Introduction

The ultraviolet (UV) irradiation inherent to sun exposure is the main factor leading to skin aging, which is also known as photoaging [1, 2]. UVB (290- to 320 nm wavelength), which can penetrate the epithelial layer and induce damage in dermal fibroblasts, plays an important role in skin photoaging [3]. Dermal fibroblasts maintain skin thickness and elasticity by producing an extracellular matrix (ECM). However, UVB irradiation can induce damage in dermal fibroblasts by producing reactive oxygen species (ROS), such as superoxide anion, hydroxyl free radicals, and hydrogen peroxide [4]; this results in decreased ECM production and remodeling [5–7]. Strategies for reducing the accumulation of intracellular ROS have been promoted to prevent UVB-induced cell death and protect skin from aging [8, 9].

Recently, stem cells have been used to treat several diseases. Adipose tissue-derived stromal/stem cells (ADSCs) are the most attractive cell type because of their ease of isolation and relative abundance [10, 11]. The therapeutic effect of ADSCs in diverse indications is attributed to their multipotent differentiation capacity and secretion of growth factors [12–14]. ADSCs have been successfully used to counteract photoaging [15–17], an effect that is likely due to their secretion of paracrine factors that act on dermal fibroblasts [18, 19].

Nanofat, an emulsified suspension derived from the processing of fat tissues with mechanical force, plays an important role in improving fat graft survival [20] and enhancing skin rejuvenation [21] and has been used to treat atrophic scars [22]. Our previous study revealed that nanofat increased dermal thickness and promoted angiogenesis [21]. Nanofat contains ADSCs and a variety of cytokines and growth factors [23]. The function of nanofat likely depends not only on the ADSCs but also on growth factors present in the emulsion. By removing the cellular and oil fractions from nanofat, we obtained a cell-free liquid suspension called fat extract (FE). FE contains multiple growth factors including insulin-like growth factor 1 (IGF-1), transforming growth factor-beta (TGF-*β*), vascular endothelial growth factor (VEGF), hepatocyte growth factor (HGF), and basic fibroblast growth factor (bFGF). The proangiogenic effects of FE have been demonstrated in our previous study [24]. We speculate that FE might also possess antioxidant activity that could prevent skin photoaging.

In the present study, we used an *in vitro* and *in vivo* photoaging model to evaluate the protective effects of FE against UVB-induced photoaging on cultured dermal fibroblasts and on the skin of nude mice.

## 2. Materials and Methods

### 2.1. Materials

Dulbecco's modified Eagle's medium (DMEM), penicillin, and streptomycin were purchased from Thermo Fisher Scientific Inc. (Waltham, MA, USA). Fetal bovine serum (FBS) was obtained from GE Healthcare Life Sciences (Logan, UT, USA). UVB light (Philips 311 nm, TL 20W/01) was from Philips Lighting Holding B.V. (Eindhoven, The Netherlands). Cell Counting Kit-8 (CCK-8) was from Beyotime Institute of Biotechnology. RNase A, propidium iodide, FITC-phalloidin, and 2′,7′-dichlorodihydrofluorescein diacetate (DCFH_2_-DA) were from Sigma-Aldrich (Merck KGaA, Darmstadt, Germany). Senescence-associated *β*-galactosidase (SA-*β*-gal) staining kit (#9860) was from Cell Signaling Technology Inc. (Danvers, MA, USA); terminal deoxynucleotidyl transferase- (TdT-) mediated dUTP-biotin nick end labeling (TUNEL) staining kit was from Roche Molecular Biochemicals (Mannheim, Germany). Anti-CD31 antibody was from Santa Cruz Biotechnology (Santa Cruz, CA, USA). Horseradish peroxidase-conjugated goat anti-mouse antibody was from Dako (Glostrup, Denmark). Anti-GPX-1 (#ab108427), anti-catalase (#ab16731), anti-SOD-2 (#ab13533), anti-SOD-1 (#ab13498), anti-COL-1 (#ab6308), and *β*-actin (#ab6276) were from Abcam (Cambridge, UK); horseradish peroxidase-conjugated goat anti-mouse secondary antibody (#115-035-062) was from Jackson ImmunoResearch Laboratories Inc. (West Grove, PA, USA).

### 2.2. FE Preparation

FE was obtained from fresh fat tissue as previously described [24]. Briefly, lipoaspirate was obtained from healthy female donors with informed consent. After washing with saline, lipoaspirate was then centrifuged to remove oil and water. The clean fat tissue was then mechanically emulsified and centrifuged to obtain the aqueous layer which was then stored at −80°C. The protein concentrations of FE were measured with a Pierce BCA protein assay kit (Thermo Fisher Scientific, Waltham, MA, USA).

### 2.3. Cell Culture

Dermal fibroblasts were obtained as previously described [25]. Cells were obtained from five donors (age, 6-12 years) who underwent a routine circumcision procedure at the Shanghai 9th People's Hospital. Cells were cultured with DMEM, supplemented with 10% FBS, 100 U/ml penicillin, and 100 *μ*g/ml streptomycin and maintained at 37°C with 5% CO_2_.

### 2.4. Cell Photoaging Model

Dermal fibroblasts were pretreated with different concentrations of FE (0, 1%, 5%, and 10%) for 24 h. Culture media were then replaced with PBS, and cells were exposed to UVB light (100 mJ/cm^2^). The media were then replaced by DMEM with 10% FBS, and cells were kept at 37°C containing 5% CO_2_. Control cells were maintained in the same conditions without UVB exposure and FE treatment.

### 2.5. Cell Proliferation Assay

Cell proliferation was detected by CCK-8 at 72 h post-UVB irradiation according to the manufacturer's instructions. Cells without FE treatment served as the control. Results are shown as a percentage relative to the control group.

### 2.6. Cell Cycle Analysis

Cell cycle analysis was performed 24 h after irradiation. Cells were collected and fixed with 70% ethanol, followed by incubation with RNase A and propidium iodide. The cell cycle was then analyzed using a flow cytometer (Beckman) with Modi Fit LT v2.0 software.

### 2.7. SA-*β*-Gal Staining

To estimate cellular aging, SA-*β*-gal staining to detect senescence was carried out 72 h postirradiation with an SA-*β*-gal staining kit as previously described [26]. After washing twice with PBS, cells were fixed and stained in accordance with the manufacturer's instructions. The number of aged cells was determined by counting SA-*β*-gal-positive cells from five random fields from each sample. Results are expressed as the percentage of aged cells among the total cells counted.

### 2.8. Cytoskeletal Protein Staining

Cells at 72 h postirradiation were collected and fixed with 4% paraformaldehyde. Fixed cells were then incubated with 0.3% Triton X-100 and incubated with FITC-phalloidin, followed by incubation with 4′,6-diamidino-2-phenylindole (DAPI). A fluorescent microscope (Olympus IX70-S1F2) was used to observe cytoskeletal proteins.

### 2.9. Measurement of Intracellular ROS

To determine intracellular ROS, DCFH_2_-DA staining was performed as previously described [27]. Cultured cells were incubated with DCFH_2_-DA after UVB irradiation, and a fluorescent microscope was used to observe ROS-dependent DCF fluorescence. Cellular DCF fluorescence was also quantified using flow cytometry analysis.

### 2.10. Skin Photoaging Model

A total of 40 six-week-old female BALB/c nude mice were used for the *in vivo* photoaging model. Protocols were approved by the Shanghai Jiao Tong University School of Medicine Animal Care and Experiment Committee. Mice were randomly divided into four groups (*n* = 10/group) as follows: the control group: mice without UVB irradiation and FE treatment, the UVB group: mice treated with UVB irradiation and subcutaneously injected with PBS, the low-dose group: mice treated with UVB irradiation and subcutaneously injected with FE (62.5 *μ*l, 300 *μ*g of total protein) once a week on the dorsal skin, and the high-dose group: mice treated with UVB irradiation and subcutaneously injected with FE (62.5 *μ*l) twice a week on the dorsal skin. UVB irradiation was performed as previously described [21]. Eight weeks after UVB irradiation with or without FE treatment, animals were sacrificed for further analyses.

### 2.11. Histological Evaluation

Samples obtained from the dorsal skin of mice were fixed with 4% paraformaldehyde and embedded in paraffin blocks from which 5 *μ*m thick sections were cut for histological evaluation. To measure the dermis thickness, hematoxylin and eosin (HE) staining and Masson's trichrome staining were performed. The dermis thickness of each sample was measured using Image-Pro Plus 6 (IPP 6) software (Media Cybernetics). To evaluate capillary density, the samples were incubated with anti-CD31 antibody, then incubated with a horseradish peroxidase-conjugated goat anti-mouse antibody. The capillary density was calculated from five randomly selected fields from each sample. To detect apoptotic cells, a TUNEL assay was performed with a TUNEL staining kit according to the manufacturer's protocol. The TUNEL-positive cells were counted in five random area images from each sample using IPP 6 software (Media Cybernetics).

### 2.12. Western Blot Analysis

Western blot analysis was performed according the standard protocol. The expression of GPX-1, catalase, SOD-2, SOD-1, and COL-1 was measured. The membranes were incubated with the following antibodies: anti-GPX-1, anti-catalase, anti-SOD-2, anti-SOD-1, anti-COL-1, and *β*-actin. Then, the membranes were incubated with horseradish peroxidase-conjugated goat anti-mouse secondary antibody. Protein bands were visualized with enhanced chemiluminescence (Pierce, Rockford, IL, USA).

### 2.13. Statistical Analysis

All data are expressed as means ± SD. One-way analysis of variance followed by Tukey's post hoc test was used for multiple comparisons. *p* < 0.05 was considered significant. All statistical analyses were performed using SPSS13.0 software (SPSS Inc., Chicago, IL, USA).

## 3. Results

### 3.1. FE Increases Cell Proliferation and Abrogates UVB Irradiation-Induced Cell Cycle Arrest

**Figure 1:**
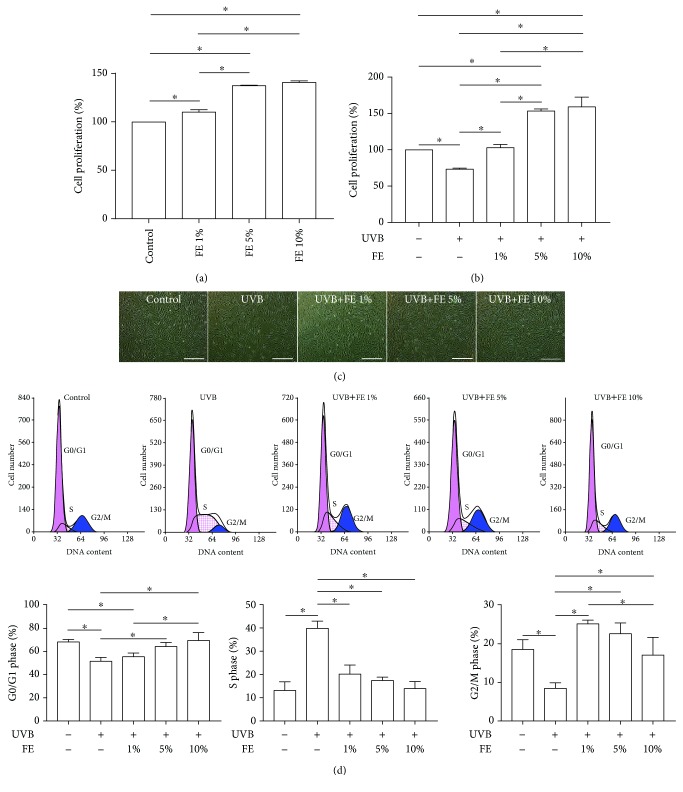
Effect of FE on the proliferation and cell cycle of human skin fibroblasts. (a) Human skin fibroblasts were treated with different concentrations of FE (0%, 1%, 5%, and 10%) for 72 h. Cell proliferation was quantified using the CCK-8 test. (b) The CCK-8 test showed cell proliferation at 72 h after cells were pretreated with or without FE (0%, 1%, 5%, and 10%) for 24 h and then exposed to UVB light. (c) Morphology of human skin fibroblasts at 72 h after UVB irradiation. Scale bar: 150 *μ*m. (d) Cell cycle analysis was carried out 24 h postirradiation. Cell cycle distribution analyzed by flow cytometry. FE treatment significantly increased cell proliferation and abrogated UVB-induced cell cycle arrest (^∗^*p* < 0.05).

FE were isolated from six donors, and the total protein concentration of FE was 4745.43 ± 751.73 *μ*g/ml. To detect the effect of FE on human skin fibroblasts, cell proliferation was measured after FE treatment either with or without UVB irradiation. As shown in Figure 1(a), in the absence of UVB irradiation, cell proliferation was significantly increased after 72 h of FE treatment in a dose-dependent manner. As expected, cell proliferation was significantly decreased after UVB irradiation, while FE abrogated the growth arrest induced by UVB (Figure 1(b)). A representative image of cellular morphology is shown in Figure 1(c); fewer cells were observed in the UVB-treated group. Cell cycle analysis showed an enrichment in the S phase and reductions in the G0/G1 and G2/M phases after UVB irradiation. However, the cell cycle profiles of FE-treated cells were similar to that of the control group (Figure 1(d)), indicating that FE prevents UVB-induced cell cycle arrest.

### 3.2. FE Prevents UVB-Induced Cell Aging

**Figure 2:**
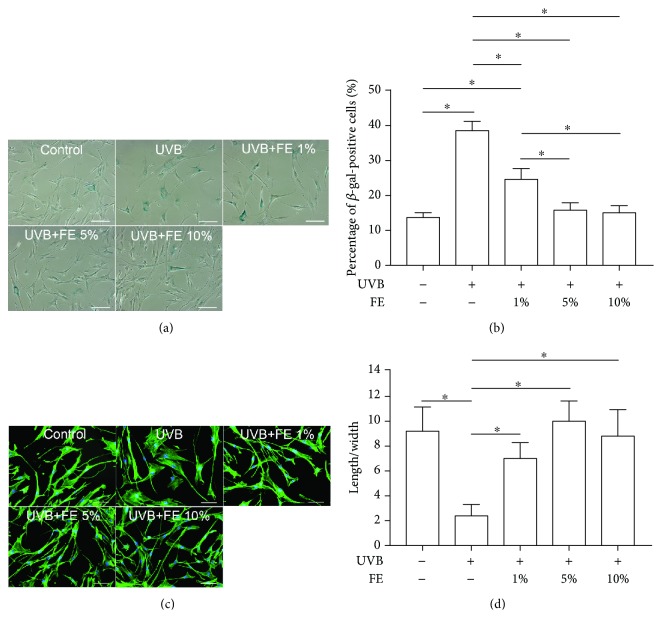
Effects of FE on SA-*β*-gal staining and morphology of human skin fibroblasts. Human skin fibroblasts were pretreated with or without FE (0%, 1%, 5%, and 10%) for 24 h, and the cells were then exposed to UVB light at a total dose of 100 mJ/cm^2^. (a) SA-*β*-gal-positive cells were present 72 h after UVB irradiation. Scale bar: 150 *μ*m. (b) The percentage of SA-*β*-gal-positive aged cells was determined by counting 200 cells per field. (c) Fluorescent labeling of cytoskeletal proteins. Scale bar: 100 *μ*m. (d) The length/width ratio of cells was determined after staining for cytoskeletal proteins. FE treatment significantly reduced the number of SA-*β*-gal-positive cells induced by UVB irradiation and preserved normal cell morphology (^∗^*p* < 0.05).

To evaluate the therapeutic effects of FE on UVB-induced cell aging, SA-*β*-gal staining was performed 72 h post-UVB and FE treatment. The number of SA-*β*-gal-positive cells was significantly increased in the UVB group but decreased in the FE-treated groups in a dose-dependent manner. However, no difference was observed between the FE 5% group and the FE 10% group (Figures 2(a) and 2(b)). Aged cells were further visualized by phalloidin staining. As shown in Figures 2(c) and 2(d), cells in the UVB group were more enlarged and flattened and had a reduced length/width ratio whereas control and FE-treated cells had a larger length/width ratio.

### 3.3. FE Reduced UVB-Induced Intracellular ROS and Promoted the Expression of GPX-1 and COL-1 *In Vitro*

**Figure 3:**
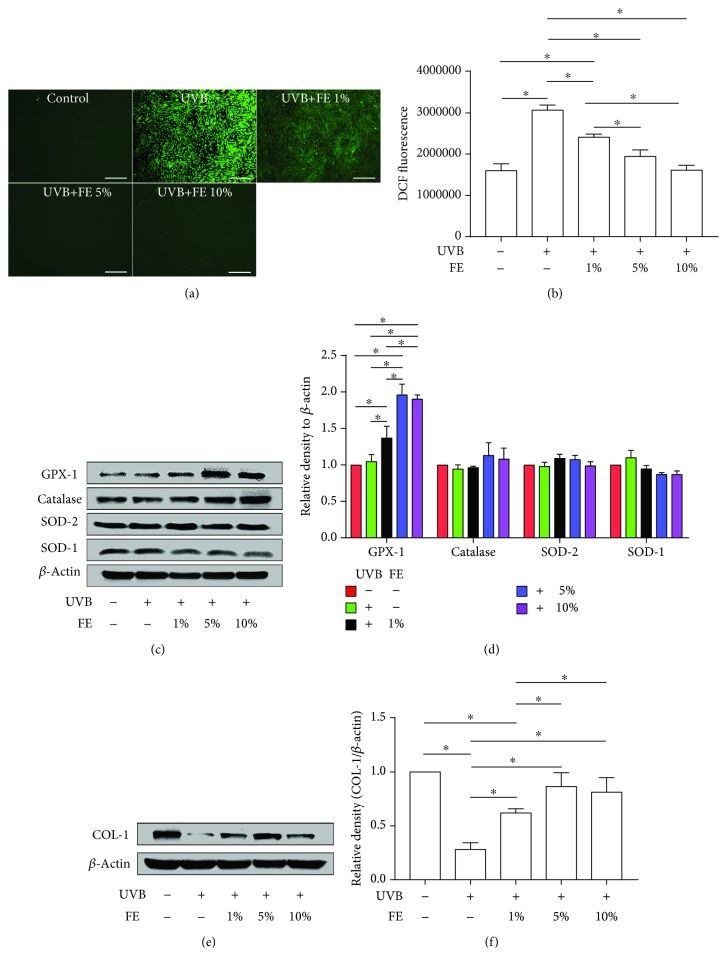
FE reduced UVB-induced intracellular ROS and promoted the expression of GPX-1 and COL-1 in human skin fibroblasts. Human skin fibroblasts were pretreated with or without FE (0%, 1%, 5%, and 10%) for 24 h, and the cells were then exposed to UVB light at a total dose of 100 mJ/cm^2^. (a) Intracellular ROS measured by DCFH_2_-DA staining 20 min after UVB irradiation. Scale bar: 500 *μ*m. (b) Intracellular ROS detected using DCFH_2_-DA staining were quantified with flow cytometry. (c, d) Western blotting analysis of GPX-1, catalase, SOD-2, and SOD-1 72 h after UVB irradiation. Representative results from three independent experiments are presented. The density values are expressed in arbitrary units. (e, f) Western blot analysis of COL-1 levels 72 h after UVB irradiation. Representative results from three independent experiments are presented. The density values are expressed in arbitrary units (^∗^*p* < 0.05).

To understand the mechanism by which FE protects cells against UVB-induced photoaging, we measured intracellular ROS levels using DCFH_2_-DA staining immediately after UVB irradiation. Fluorescent microscopy revealed a higher level of ROS in the UVB group, while lower levels were observed in the FE-treated groups and the control group (Figure 3(a)). The result was confirmed by analyzing the DCF fluorescence intensity using a flow cytometer (Figure 3(b)). To further delineate the mechanism underlying FE-dependent reduction of ROS, we measured the major antioxidant proteins GPX-1, catalase, SOD-2, and SOD-1 by western blot analysis. No differences were found in the control group and the UVB group, while the expression of GPX-1, one of the most important cellular antioxidant enzymes, was significantly increased in the FE-treated groups compared with the UVB group; the levels of SOD-1, SOD-2, and catalase were not increased under these conditions (Figures 3(c) and 3(d)). Meanwhile, the expression of COL-1, the most important ECM protein produced by dermal fibroblasts, was downregulated in the UVB group compared with the control group but upregulated in the FE-treated groups (Figures 3(e) and 3(f)).

### 3.4. Antiaging Effects of FE on the Skin of UVB-Irradiated Nude Mice

**Figure 4:**
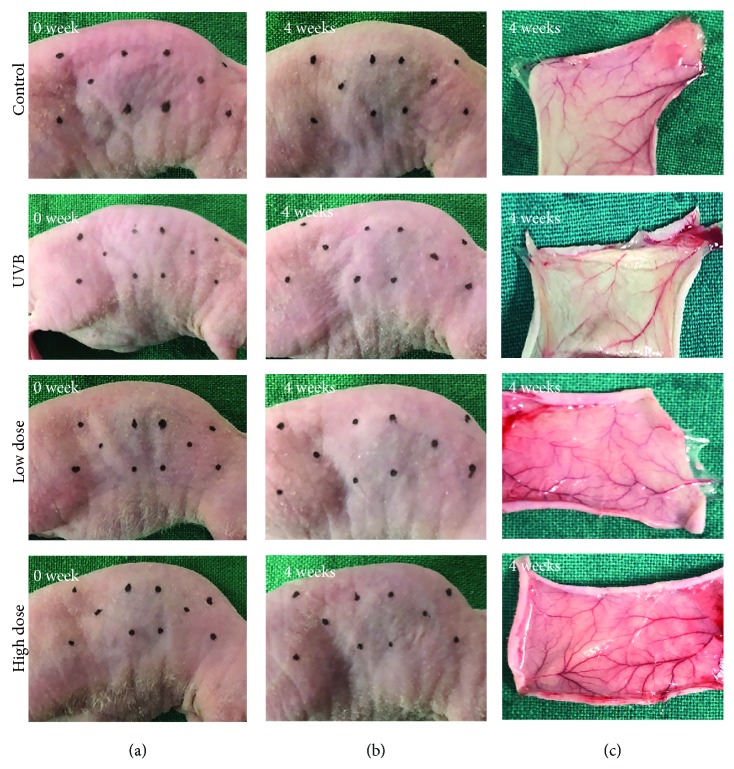
Macroscopic observation after UVB irradiation and FE treatment for 4 weeks. (a, b) There were no differences in the appearance of the dorsal skin between the UVB group and FE treatment groups. (c) No FE residue was observed in the dorsal region where injection was performed, but more blood vessels were observed in the FE treatment group than in the UVB group.

**Figure 5:**
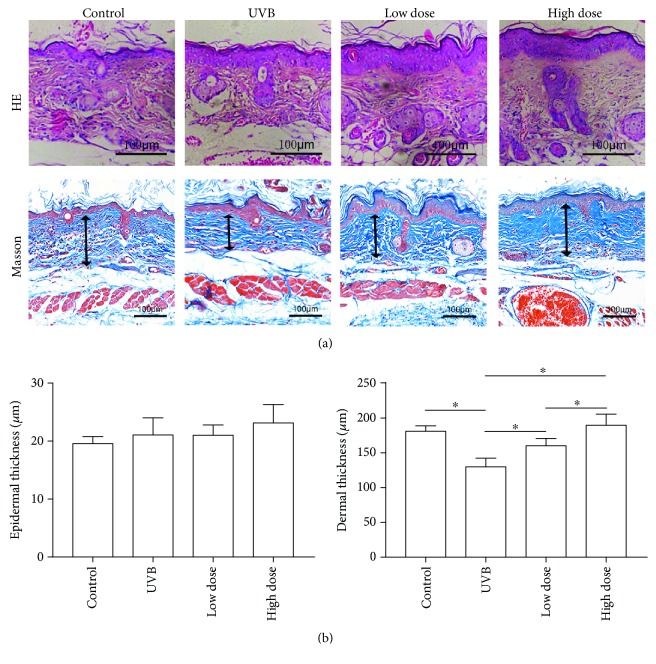
Effect of FE on UVB-induced alterations of the epidermis and dermis in mice. (a) HE and Masson's trichrome staining were performed after FE treatment for 8 weeks. The arrows indicate the dermal portion of the skin. Scale bar: 100 *μ*m. (b) Quantitative analysis of the epidermal and the dermal skin thickness. FE treatment significantly increased the thickness of the dermal portion of the skin (^∗^*p* < 0.05).

**Figure 6:**
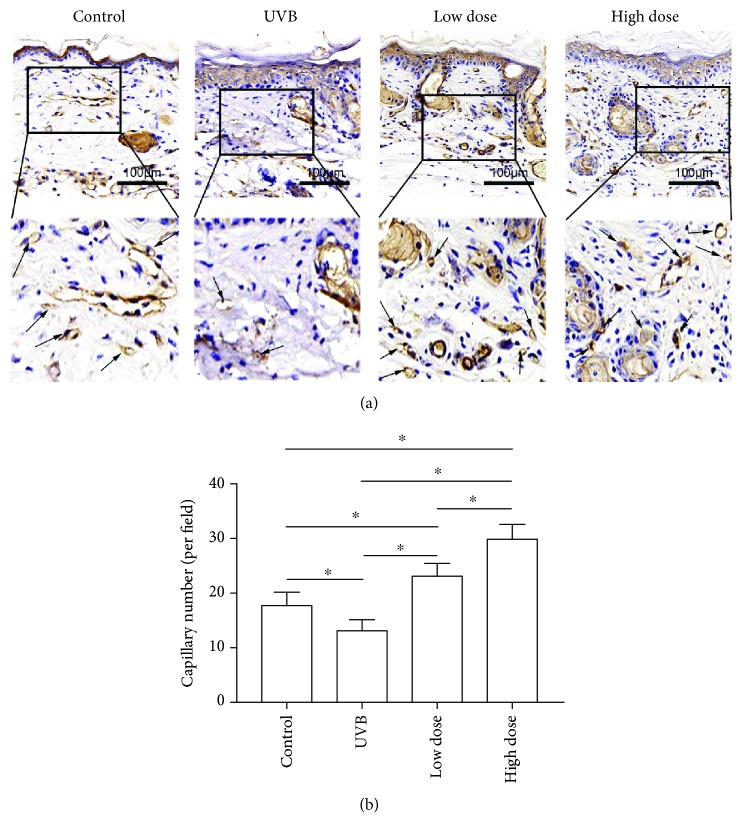
Effect of FE on angiogenesis in mice. (a) Anti-CD31 staining was performed after FE treatment for 8 weeks. The arrows indicate blood vessels. Scale bar: 100 *μ*m. (b) FE treatment significantly increased vascular density in the dermis (^∗^*p* < 0.05).

We next performed an *in vivo* study to confirm the therapeutic effects of FE on UVB-induced skin aging in nude mice. Animals were UVB-irradiated and treated with or without FE for up to 8 weeks. There were no gross differences in the appearance of the dorsal skin between the UVB group and the FE groups at 4 weeks (Figures 4(a) and 4(b)) or 8 weeks (data not shown), and no FE residue was observed at the injection site. However, more blood vessels in the skin were observed in the FE groups (Figure 4(c)). At 8 weeks posttreatment, the thickness of the epidermal layer was slightly increased, while the dermal layer was significantly decreased in the UVB group compared with the control group (without UVB irradiation and FE treatment; Figures 5(a) and 5(b)). In the FE-treated groups, the dermal layer thickness was increased. The dermal layer was also thicker in the high-dose FE group than in the low-dose group (Figures 5(a) and 5(b)). Next, the capillaries in the dermis were evaluated using anti-CD31 staining. Capillary density was lower in the UVB group compared with the control group but was higher in the FE-treated groups. Again, more vessels were observed in the high-dose FE group than in the low-dose group (Figure 6).

### 3.5. FE Reduces ROS-Induced Cell Apoptosis and Promotes GPX-1 and COL-1 Expression *In Vitro*

**Figure 7:**
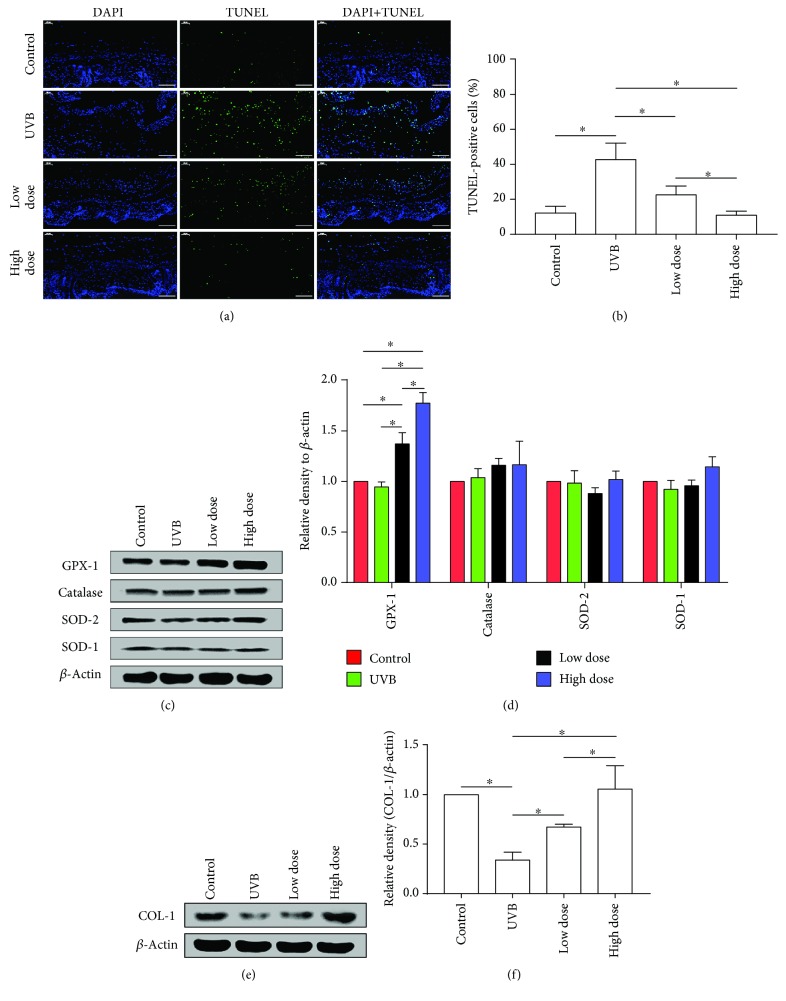
FE reduced ROS-induced cell apoptosis and promoted the expression of GPX-1 and COL-1 in mice. (a) Fluorescence microscopy images and (b) quantification (%) of apoptotic cells following labeling with a TUNEL staining kit. Scale bar: 100 *μ*m. (c, d) Western blotting analysis of GPX-1, catalase, SOD-2, and SOD-1 in mice after FE treatment for 8 weeks. Representative results from three independent experiments are presented. The density values are expressed in arbitrary units. (e, f) Western blotting analysis of COL-1 in mice after FE treatment for 8 weeks. Representative results from three independent experiments are presented. The density values are expressed in arbitrary units (^∗^*p* < 0.05).

Since intracellular ROS can induce apoptosis [28], we analyzed the number of apoptotic cells in mice skin using TUNEL staining. More TUNEL-positive cells were observed in the UVB group, suggesting that UVB irradiation induces apoptosis. FE treatment significantly decreased the number of TUNEL-positive cells compared with the UVB group, and this effect was dose-dependent (Figures 7(a) and 7(b)). We also evaluated the expression of GPX-1, catalase, SOD-2, SOD-1, and COL-1 in the skin by western blot. GPX-1 expression was not changed in the UVB group compared with the control group but was upregulated in the FE-treated groups (Figures 7(c) and 7(d)). The expression of SOD-1, SOD-2, and catalase was not increased. While COL-1 expression was decreased in the UVB group, it was restored in the FE groups in a dose-dependent manner (Figures 7(e) and 7(f)).

## 4. Discussion

Photoaging is commonly induced by UV irradiation, which stimulates the expression of intracellular ROS. The increased ROS inhibit the proliferation of dermal fibroblasts and the production and remodeling of ECM [5]. In the current study, we demonstrated that FE reduced the accumulation of intracellular ROS induced by UVB treatment, partially via the upregulation of the antioxidant enzyme, GPX-1. The protective effects were observed in both cultured dermal fibroblasts and the skin of nude mice.

In cultured dermal fibroblasts, expression of intracellular ROS was increased by UVB irradiation, resulting in reduced proliferation, cell cycle arrest, increased senescence, and inhibition of ECM synthesis. FE treatment significantly attenuated the UVB-dependent increase in intracellular ROS, abrogated UVB-induced cell cycle arrest, reduced senescence, and restored COL-1 production by fibroblasts. The regulation of antioxidant enzyme expression after FE treatment indicated that FE likely works through upregulation of GPX-1. FE contains a variety of growth factors, including TGF-*β*, VEGF, HGF, bFGF, and IGF-1 [24], and the antioxidant effects of these growth factors have been demonstrated in several models. For example, IGF-1 exerts an antioxidant effect on fibroblasts and attenuates H_2_O_2_-induced oxidative stress via the PI3K pathway [29, 30]. HGF can protect cultured rat mesangial cells against oxidative stress via inhibition of PKA and activation of PKG [31]. Although the underlying mechanisms remain to be determined, it is clear from our results that FE treatment is a selective activator of GPX-1 and does not appear to engage the signaling pathways that increase the activities of SOD-1, SOD-2, or catalase. It has been reported that GPX-1 was regulated through the nuclear factor erythroid 2-related factor 2 (Nrf2) pathway, while SODs were regulated through the Sirt1/FOXO pathway [32, 33]. The detailed mechanism is investigating in the future.

Besides the antioxidant pathway, the presence of a variety of growth factors in FE suggests that it may work together with other pathways *in vivo*. The first pathway is the stimulation of new blood vessel formation in the dermis. We observed an increased number of capillaries after FE treatment, which is in accordance with our previous studies [24]. The second pathway engages antiapoptotic activity, as fewer apoptotic cells were observed in FE-treated skin. Although the antiapoptotic activities of VEGF, HGF, and IGF-1 have been demonstrated in different cell types [34, 35], the mechanism by which FE protects against cell death is not clear. We speculate that FE may exert its effects partially through the antioxidant pathway. The third pathway is the stimulation of cell proliferation and ECM production. It is not surprising that FE contains a variety of mitogens. The effects of a single growth factor on dermal fibroblasts, such as HGF, VEGF, and bFGF have been well demonstrated in many studies. The antioxidant, antiapoptotic, and proangiogenic activities of FE could be explained by the presence of a variety of growth factors, which indicates that the level of growth factors could be a good parameter for quality control of FE preparation.

High-dose FE treatment was more effective than low-dose treatment, suggesting that multiple injections are preferable. Compared with nanofat, FE is cell- and oil-free, which addresses any concerns related to using cellular therapies. In addition, the shelf life of FE is much longer than that of nanofat, which may facilitate clinical applications that require multiple administrations of the extract. Banking autologous ADSCs and FE from fat tissue that is removed from patients who undergo liposuction will generate a valuable source of therapeutic material for future needs. In the current study, only lipoaspirates from healthy female donors have been analyzed. FE prepared from a male donor is worth investigating in the future.

## 5. Conclusion

In summary, our study demonstrates the protective effects of FE against UVB-induced skin photoaging. This is mediated by the antioxidant, antiapoptotic, and proangiogenic activities of FE, which may be a potential antiphotoaging reagent for skin rejuvenation.

## Data Availability

The data used to support the findings of this study are included within the article.

## References

[1] Gilchrest B. A. (2013). Photoaging. *The Journal of Investigative Dermatology*.

[2] Kammeyer A., Luiten R. M. (2015). Oxidation events and skin aging. *Ageing Research Reviews*.

[3] Lee Y. R., Noh E. M., Jeong E. Y. (2009). Cordycepin inhibits UVB-induced matrix metalloproteinase expression by suppressing the NF-*κ*B pathway in human dermal fibroblasts. *Experimental & Molecular Medicine*.

[4] Poon F., Kang S., Chien A. L. (2015). Mechanisms and treatments of photoaging. *Photodermatology, Photoimmunology & Photomedicine*.

[5] Brenneisen P., Sies H., Scharffetter-Kochanek K. (2002). Ultraviolet-B irradiation and matrix metalloproteinases: from induction via signaling to initial events. *Annals of the New York Academy of Sciences*.

[6] Rittié L., Fisher G. J. (2002). UV-light-induced signal cascades and skin aging. *Ageing Research Reviews*.

[7] Rabe J. H., Mamelak A. J., McElgunn P. J. S., Morison W. L., Sauder D. N. (2006). Photoaging: mechanisms and repair. *Journal of the American Academy of Dermatology*.

[8] Stern R. S. (2004). Treatment of photoaging. *The New England Journal of Medicine*.

[9] Petruk G., del Giudice R., Rigano M. M., Monti D. M. (2018). Antioxidants from plants protect against skin photoaging. *Oxidative Medicine and Cellular Longevity*.

[10] Harasymiak-Krzyżanowska I., Niedojadło A., Karwat J. (2013). Adipose tissue-derived stem cells show considerable promise for regenerative medicine applications. *Cellular & Molecular Biology Letters*.

[11] Gimble J. M., Katz A. J., Bunnell B. A. (2007). Adipose-derived stem cells for regenerative medicine. *Circulation Research*.

[12] Sun M., Wang S., Li Y. (2013). Adipose-derived stem cells improved mouse ovary function after chemotherapy-induced ovary failure. *Stem Cell Research & Therapy*.

[13] Yang J., Zhang Y., Zang G. (2018). Adipose-derived stem cells improve erectile function partially through the secretion of IGF-1, bFGF, and VEGF in aged rats. *Andrology*.

[14] Gong B., Dong Y., He C. (2019). Intravenous transplants of human adipose-derived stem cell protect the rat brain from ischemia-induced damage. *Journal of Stroke and Cerebrovascular Diseases*.

[15] Xu X., Wang H. Y., Zhang Y. (2014). Adipose-derived stem cells cooperate with fractional carbon dioxide laser in antagonizing photoaging: a potential role of Wnt and *β*-catenin signaling. *Cell & Bioscience*.

[16] Jeong J. H., Fan Y., You G. Y., Choi T. H., Kim S. (2015). Improvement of photoaged skin wrinkles with cultured human fibroblasts and adipose-derived stem cells: a comparative study. *Journal of Plastic, Reconstructive & Aesthetic Surgery*.

[17] Son W. C., Yun J. W., Kim B. H. (2015). Adipose-derived mesenchymal stem cells reduce MMP-1 expression in UV-irradiated human dermal fibroblasts: therapeutic potential in skin wrinkling. *Bioscience, Biotechnology, and Biochemistry*.

[18] Kim W.-S., Park B. S., Sung J. H. (2009). Protective role of adipose-derived stem cells and their soluble factors in photoaging. *Archives of Dermatological Research*.

[19] Kim W. S., Park B. S., Park S. H., Kim H. K., Sung J. H. (2009). Antiwrinkle effect of adipose-derived stem cell: activation of dermal fibroblast by secretory factors. *Journal of Dermatological Science*.

[20] Yu Q., Cai Y., Huang H. (2018). Co-transplantation of nanofat enhances neovascularization and fat graft survival in nude mice. *Aesthetic Surgery Journal*.

[21] Xu P., Yu Q., Huang H., Zhang W. J., Li W. (2018). Nanofat increases dermis thickness and neovascularization in photoaged nude mouse skin. *Aesthetic Plastic Surgery*.

[22] Gu Z., Li Y., Li H. (2018). Use of condensed nanofat combined with fat grafts to treat atrophic scars. *JAMA Facial Plastic Surgery*.

[23] Liang Z. J., Lu X., Li D. Q. (2018). Precise intradermal injection of nanofat-derived stromal cells combined with platelet-rich fibrin improves the efficacy of facial skin rejuvenation. *Cellular Physiology and Biochemistry*.

[24] Yu Z., Cai Y., Deng M. (2018). Fat extract promotes angiogenesis in a murine model of limb ischemia: a novel cell-free therapeutic strategy. *Stem Cell Research & Therapy*.

[25] Chen F. G., Zhang W. J., Bi D. (2007). Clonal analysis of nestin^−^ vimentin^+^ multipotent fibroblasts isolated from human dermis. *Journal of Cell Science*.

[26] Dimri G. P., Lee X., Basile G. (1995). A biomarker that identifies senescent human cells in culture and in aging skin *in vivo*. *Proceedings of the National Academy of Sciences of the United States of America*.

[27] Jia C., Lu Y., Bi B. (2017). Platelet-rich plasma ameliorates senescence-like phenotypes in a cellular photoaging model. *RSC Advances*.

[28] Nishi K., Iwaihara Y., Tsunoda T. (2017). ROS-induced cleavage of NHLRC2 by caspase-8 leads to apoptotic cell death in the HCT116 human colon cancer cell line. *Cell Death & Disease*.

[29] Baregamian N., Song J., Jeschke M. G., Evers B. M., Chung D. H. (2006). IGF-1 protects intestinal epithelial cells from oxidative stress-induced apoptosis. *Journal of Surgical Research*.

[30] Rahman Z. A., Soory M. (2006). Antioxidant effects of glutathione and IGF in a hyperglycaemic cell culture model of fibroblasts: some actions of advanced glycaemic end products (AGE) and nicotine. *Endocrine, Metabolic & Immune Disorders Drug Targets*.

[31] Hui L., Hong Y., Jingjing Z., Yuan H., Qi C., Nong Z. (2010). HGF suppresses high glucose-mediated oxidative stress in mesangial cells by activation of PKG and inhibition of PKA. *Free Radical Biology & Medicine*.

[32] Ooi B., Goh B., Yap W. (2017). Oxidative stress in cardiovascular diseases: involvement of Nrf2 antioxidant redox signaling in macrophage foam cells formation. *International Journal of Molecular Sciences*.

[33] Zhang W., Huang Q., Zeng Z., Wu J., Zhang Y., Chen Z. (2017). Sirt1 inhibits oxidative stress in vascular endothelial cells. *Oxidative Medicine and Cellular Longevity*.

[34] Chimenti I., Smith R. R., Li T. S. (2010). Relative roles of direct regeneration versus paracrine effects of human cardiosphere-derived cells transplanted into infarcted mice. *Circulation Research*.

[35] Zhang Z., Zhao C., Liu B. (2014). Inositol pyrophosphates mediate the effects of aging on bone marrow mesenchymal stem cells by inhibiting Akt signaling. *Stem Cell Research & Therapy*.

